# Surgical outcomes of borderline breast lesions detected by needle biopsy in a breast screening program

**DOI:** 10.1186/1477-7819-8-78

**Published:** 2010-09-08

**Authors:** Karen M Flegg, Jeffrey J Flaherty, Anne M Bicknell, Sanjiv Jain

**Affiliations:** 1 Australian National University, Medical School. The Canberra Hospital, Building 4, PO Box 11, Woden, ACT, 2606, Australia; 2 BreastScreen ACT&SENSW. 1 Moore Street, Canberra, ACT, 0200, Australia; 3 The Canberra Hospital, Woden, ACT, 2606, Australia

## Abstract

**Background:**

The Australian Capital Territory and South East New South Wales branch of BreastScreen Australia (BreastScreen ACT&SENSW) performs over 20,000 screening mammograms annually. This study describes the outcome of surgical biopsies of the breast performed as a result of a borderline lesion being identified after screening mammography and subsequent workup.

A secondary aim was to identify any parameters, such as a family history of breast cancer, or radiological findings that may indicate which borderline lesions are likely to be upgraded to malignancy after surgery.

**Methods:**

From a period of just over eight years, all patients of BreastScreen ACT&SENSW who were diagnosed with a borderline breast lesion were identified. These women had undergone needle biopsy in Breastscreen ACT&SENSW and either atypical ductal hyperplasia (ADH), flat epithelial atypia (FEA), atypical lobular hyperplasia (ALH), radial scar/complex sclerosing lesion, papillary lesion, mucocoele-like lesion (MLL) or lobular carcinoma in situ (LCIS) was found. Final outcomes for each type of borderline lesion after referral for surgical biopsy were recorded and analysed. Results of the surgical biopsy were compared to the type of needle biopsy and its result, radiological findings and family history status.

**Results:**

Of the 94 surgical biopsies performed due to the presence of a borderline breast lesion, 20% showed benign pathology, 55% remained as borderline lesions, 17% showed non-invasive malignancy and 7% showed invasive malignancy. VALCS biopsy was the most common needle biopsy method used to identify the lesions in this study (76%). Malignant outcomes resulted from 24% of the surgical biopsies, with the most common malignant lesion being non-comedo ductal carcinoma *in situ *(DCIS). The most common borderline lesion for which women underwent surgical biopsy was ADH (38%). Of these women, 22% were confirmed as ADH on surgical biopsy and 47% with a malignancy.

**Conclusions:**

Further research is required to determine whether characteristics of the mammographic lesion (particularly calcification patterns), the area targeted for biopsy and number of core samples retrieved, can indicate a closer correlation with eventual pathology. This study identified no findings in the diagnostic assessment that could exclude women with borderline lesions from surgical biopsy.

## Background

The Australian Capital Territory and South East New South Wales (BreastScreen ACT&SENSW) branch of BreastScreen Australia performs over 20,000 screening mammograms annually[1]. The program specifically targets women aged 50 to 69 years and also allows access to women from 40-49 and 70 and over. Screening consists of two mammography views of each breast, a mediolateral oblique and a cranio-caudal view. Films are double read by two radiologists operating independently and if an abnormality is detected by mammography, patients are recalled for workup, often including fine needle biopsy (FNB) and/or a core needle biopsy (CNB) guided by ultrasound (14-16 gauge), or a vacuum assisted large core (11 gauge) stereotactic (VALCS) biopsy.

There are a number of lesions which, when detected using a needle biopsy cause diagnostic uncertainty. These so called borderline breast lesions, are lesions which may coexist with breast malignancy, or lie on a spectrum of pathological entities which are difficult to distinguish from malignant lesions[2,3]. The borderline lesions recognised in this study are ADH[4], LCIS, ALH[5], papillary lesions, FEA[6], MLL[7] and complex sclerosing lesions/radial scars[8]. Currently, at BreastScreen ACT&SENSW, if FNB or CNB demonstrate borderline lesions the patient is referred for a diagnostic surgical biopsy to exclude the possibility of a closely situated carcinoma.

Borderline lesions are a relatively rare occurrence and for this reason there is little data in the literature from which pathologists, breast physicians and surgeons can construct management plans with an evidence based focus[2]. The primary aim of this study was to describe how many malignancies are revealed when surgical biopsy is performed due to the presence of a borderline lesion on needle biopsy in an Australian breast screening program. The secondary aim was to identify any parameters, such as a family history of breast cancer, or findings during radiological assessment that may indicate which lesions are likely to be upgraded to malignancy. These findings could result in recommendations that would lead to a reduction in the number of women that undergo a purely diagnostic surgical biopsy.

## Methods

During the period August 1998 to November 2006, a total of 167 women underwent a diagnostic open surgical biopsy as a result of a screen detected mammographic abnormality at BreastScreen ACT&SENSW. Patients were selected for inclusion in this study if they were referred for surgical biopsy because their FNB or CNB showed a borderline lesion, namely, ADH, FEA, ALH, radial scar/complex sclerosing lesion, papillary lesion, MLL or LCIS. This resulted in a total of 94 women with borderline lesions being included in this study.

The medical records of the included women were examined to determine the results of their mammogram, ultrasound, and family history status. Family history status was established using the three categories of risk used by the National Breast and Ovarian Cancer Centre (NBOCC)[9]. Results of their surgical biopsy were compared to their needle biopsy result and the type of needle biopsy that was done. All pathology was reviewed by two independent pathologists experienced in breast pathology, in line with the normal protocol at BreastScreen ACT&SENSW.

Fibroadenomas/phyllodes lesions were also examined but for the purposes of this study, were not included as borderline lesions. At BreastScreen ACT&SENSW fibroepithelial lesions are excised if the imaging suggests a suspicious pattern or the distinction cannot be made between a fibroadenoma and a phyllodes tumour.

Ethics approval was given by the human research ethics committee of Australian Capital Territory (ACT) Health and a sub-committee representing the Australian National University (ANU) Medical School on behalf of the ANU human research ethics committee.

## Results

During the study period of just over eight years, there were 94 borderline lesions identified on needle biopsy that were recommended for a surgical biopsy. Vacuum assisted large core stereotactic guided core needle (VALCS) biopsy was the most common needle biopsy method used to identify the lesions in this study (76%).

Malignant outcomes were revealed in 24% (n = 23) of the surgical biopsies, with the most common malignant lesion being non-comedo ductal carcinoma *in situ *(DCIS). (Table 1) Of the borderline lesions identified by VALCS biopsy, malignancy was found in 31% of cases. None of the lesions identified using US guided CNB showed malignancy on open biopsy. When a borderline lesion was diagnosed on FNB only, then open biopsy showed a malignant lesion in 10% (n = 1) of cases.

**Table 1:** Needle biopsy diagnosis* compared to results of surgical biopsy.

Needle Biopsy Diagnosis*	N =	Surgical Biopsy Diagnosis		
		
		**Non-malignant**‡	DCIS	Invasive Malignancy
ADH	36	19(53%) §	13(36%)	4(11%)

ALH	4	4(100%)		

LCIS	5	3(60%)	1(20%)	1 (20%)

Radial Scar †	18	18(100%)||		

FEA	5	3(60%)	1(20%)	1 (20%)

MLL	3	3(100%) ¶		

Papillary lesion	23 **	21(91%)	1(4%) ††	1(4%) ‡‡

**Total**	**94**	**71(76%)**	**16(17%)**	**7(7%)**

* biopsy type = Vacuum assisted large core stereotactic guided core needle biopsy (VALCS) unless stated otherwise

† Radial Scar represents both radial scars and complex sclerosing lesions.

‡ Non-Malignant lesions include borderline lesions and benign lesions (ductal hyperplasia with no atypia, sclerosing adenosis, fibroadenoma, fibrocystic change and scar tissue).

§ biopsy type on one lesion by Ultrasound guided core needle biopsy (USCNB)

|| biopsy type on four lesions by USCNB

¶ biopsy type on one lesion USCNB and on two lesions Fine needle biopsy (FNB)

** biopsy type on seven lesions USCNB and on eight lesions FNB

†† biopsy type VALCS only

‡‡ biopsy type FNB only

ADH = Atypical ductal hyperplasia; ALH = Atypical lobular hyperplasia; FEA = Flat epithelial atypia; LCIS = Lobular carcinoma *in situ*; MLL = Mucocoele-like lesions;

The most common borderline lesion for which women underwent surgical biopsy was ADH (38%). Of these 36 women, 22% (n = 8) were confirmed as ADH on surgical biopsy and 47% (n = 17) with a malignancy. The final pathology of the others originally thought to have ADH showed ductal hyperplasia with no atypia (n = 4), ALH (n = 1), radial scar (n = 1), FEA (n = 2), fibrocystic change (n = 2) and benign scar tissue (n = 1).

All radial scars had a benign outcome after surgical biopsy, with 72% having no change to their diagnosis, while the remainder consisted of ADH (n = 1), fibrocystic change (n = 3) and papillary lesions (n = 1).

The radiographic abnormalities for which the women underwent a needle biopsy are shown in table 2. Needle biopsies performed for calcifications, stellate lesions and discrete opacities showed malignancy following surgical biopsy in 33%, 17% and 16% of cases respectively. The radiological findings are shown with the pre- and post surgical diagnosis to highlight the information at different stages of the diagnostic triple test. Its is also useful because although the definitive diagnosis may change from a borderline to malignant lesion, the borderline lesion was still an identified pathological process with associated mammographic findings.

**Table 2:** Radiological findings for pre- and post- surgical diagnosis of borderline lesions.

Diagnosis	Mammogram Findings n = (%)						
	
	Calcification	Stellate lesion	Discrete opacity	Multiple opacities	Architectural distortion	Non specific density	Total
Total	51 (54%)	18 (19%)	19 (20%)	1 (1%)	4 (4%)	1 (1%)	94

**Mammogram findings by NEEDLE BIOPSY diagnosis**							

ADH	33 (92%)	2 (6%)			1 (3%)		36

ALH	3 (75%)				1 (25%)		4

LCIS	4 (80%)		1 (20%)				5

Radial Scar*	2 (11%)	14 (78%)			2 (11%)		18

FEA	4 (80%)	1 (20%)					5

MLL			3 (100%)				3

Papillary Lesion	5 (22%)	1 (4%)	15 (65%)	1 (4%)		1 (4%)	23

**Mammogram findings by SURGICAL BIOPSY diagnosis**							

ADH	10 (91%)	1 (9%)					11

ALH	4 (100%)						4

LCIS	1 (100%)						1

Radial Scar*		12 (67%)		1 (6%)	4 (22%)	1 (6%)	18

FEA	2 (100%)						2

MLL			2 (100%)				2

Papillary Lesion	2 (14%)	1 (7%)	11 (79%)				14

Other benign dysplasia^†^	15 (79%)	1 (5%)	3 (16%)				19

DCIS	13 (81%)	1 (6%)	2 (13%)				16

Invasive Malignancy	4 (57%)	2 (29%)	1 (14%)				7

* Radial Scar represents both radial scars and complex sclerosing lesions.

† Other benign dysplasia includes lesions such as ductal hyperplasia with no atypia (n = 8), sclerosing adenosis (n = 2), fibroadenoma (n = 1), fibrocystic change (n = 7) and scar tissue (n = 1).

ADH, Atypical ductal hyperplasia; ALH, Atypical lobular hyperplasia; FEA, Flat epithelial atypia; FNB, Fine needle biopsy; LCIS, Lobular carcinoma *in situ*; MLL, Mucocele-like lesions; US CNB, Ultrasound guided core needle biopsy; VALCS, Vacuum assisted large core stereotactic guided core needle biopsy.

Only one woman, of the 23 with malignant diagnoses, was in a high risk group based on family history (ADH on needle biopsy and eventually diagnosed with a tubular carcinoma), five women were in the moderate risk group and 17 had no increased risk compared to the general population.

Of women who had a surgical biopsy, 59% had an ultrasound performed after the initial abnormality was detected by mammography. The ultrasound findings were extremely variable and hence the data is not shown. However, all patients who had a solid lesion designated as likely to be benign on the ultrasound (n = 10) were diagnosed with a non-malignant lesion after a surgical biopsy.

## Discussion

The 'triple test' diagnostic process that involves a clinical examination, radiological assessment and a needle biopsy is 99.6% sensitive, and has 93% specificity for identifying a breast malignancy[10]. However, surgical biopsy is often required for complete assessment of borderline lesions. This study looked at the outcomes of all surgical biopsies performed due to the presence of borderline lesions on CNB or FNB, during a period of just over eight years, at BreastScreen ACT&SENSW. In this study 24% of women who underwent a surgical biopsy were found to have an invasive breast malignancy or DCIS which can be compared to reported malignancy rates for borderline lesions as high as 35% in non-screening populations[3].

ADH was the most common lesion for which women were referred for a surgical biopsy and previous studies which were not limited to screening populations, report comparable rates of malignant diagnosis (31-48% as compared with 47% in this study) after surgical excision of ADH with some smaller samples report rates ranging from 10-60%[3,4,11]. The concerns with ADH are that it not only coexists with malignancy, it is a non-obligate precursor to breast cancer[4,11-15]. Moreover, it is difficult to distinguish ADH from a low grade DCIS and in sampling a lesion using CNB, any ADH seen on histological assessment may represent only part of a lesion which also contains DCIS.

It is of interest that 11 of the ADH lesions identified at needle biopsy showed no ADH or malignant lesion on surgical biopsy. The explanation for this may be that the area of abnormality in these lesions was either completely excised during the needle biopsy procedure, or the sections of the surgical biopsy specimen examined by the pathologist may not have been sufficiently representative of the entire lesion.

Malignancy rates in the order of 30% with FEA after surgical excision have been reported[16] and we found a similarly high rate of malignancy in the women with FEA (40%). FEA lesions are excised because they have been shown to coexist with carcinoma and the atypia falls in a spectrum of pathology that encompasses ADH and DCIS[6,17].

The lobular neoplasias, ALH and LCIS are less common and 17-44% are reported to be associated with malignancy[5,8,14]. In our study none of the women with ALH, but 40% of women with LCIS on needle biopsy had a malignancy. The presence of lobular neoplasia is a risk factor for the development of breast cancer in either breast[18]. If the sample size of our study were larger we might have expected rates of malignancy after excision of ALH lesions, to be closer to those published by others which ranged from 14-25%[5,11,14]. The same studies report a malignant diagnosis of 25-33% of women initially diagnosed with LCIS[5,11,14].

In this study none of the radial scar diagnoses at needle biopsy were shown to have a malignancy at surgery. Radial scars have previously been shown to have a significant rate (up to 25%) of associated malignancy[3,19]. For this reason, even though this study showed no upgrade from the diagnosis of a radial scar to a malignancy we recommend that lesions designated as radial scar or a complex sclerosing lesion continue to be excised. Evidence also supports that it is an independent risk factor for breast cancer which warrants aggressive screening[8,20,21].

Only three MLL were identified in the current study and none were associated with a malignant surgical specimen. One small study found malignancy in up to 30% of MLL,[7] however all of these were associated with ADH and would have been included in the ADH group in our study. Because, MLL are difficult to distinguish from mucinous carcinoma excision is still recommended[2].

It is reported that 14% of atypical papillomata and 23% of all papillary lesions are associated with either DCIS or papillary carcinoma[3,8]. ADH and lobular neoplasia are also known to commonly coexist with papillomas[22]. In our study 9% of papillary lesions were found to have a malignant surgical specimen. The papillary lesions that were found to be malignant were both discrete opacities on mammograms, it is of note that the non-comedo DCIS after excision was identified as a papillary lesion after VALCS biopsy, and the intraduct papillary carcinoma was initially picked up on FNA.

CNB guided by ultrasound was the only method in which no borderline lesions were subsequently found to be malignant. Surgical biopsy done on borderline lesions identified on cytological assessment after FNB showed 10% to be malignant. These are not unexpected findings as the majority of lesions biopsied by VALCS were calcifications compared to the lesions biopsied by FNB and CNB which were more likely to be mammographic and/or sonographic densities. Almost one third of borderline lesions identified by VALCS were eventually diagnosed with a breast malignancy. Borderline lesions may coexist with and be difficult to distinguish from malignant lesions[2,3]. It is for this reason that a high index of suspicion for co-existing malignant disease should be entertained and that surgical excision should be undertaken to exclude such. This study also looked at identifying further criteria that might indicate which borderline lesions were more likely to be diagnosed as a malignancy. Our results and other studies have reported variable findings on mammography and ultrasound[3,14,23,24].

## Conclusions

The strength of this study comes from the relatively large sample of borderline lesions over a period of more than eight years. The main limitation is the small numbers of each type of lesion. With the exception of ADH, borderline lesions are rare findings.

This is a study of a screening population; hence the data is not confounded by clinical findings that may contribute to the decision for surgical excision. A multicentre study is recommended to increase the sample size in order to enable further review of the less common lesions. Also, this study did not look at long term follow up of these women; further research could contribute more information about the longer term outcomes after a biopsy shows a borderline lesion.

Further research is required to determine whether characteristics of the mammographic lesion (particularly calcification patterns), the area targeted for biopsy and number of core samples retrieved, can indicate a closer correlation with eventual pathology. In the present study, there were only a small number of malignancies associated with a significant family history and subsequent increased risk of breast cancer. As a result it should not influence the approach taken to borderline lesions in a breast screening program.

In conclusion, although surgical biopsies are invasive procedures with associated risk, they remain an important part of the diagnostic process. We recommend that for diagnostic certainty, surgical biopsies should continue to be performed after a needle biopsy identifies a borderline lesion.

## Competing interests

AMB is employed as the Clinical Director of Breastscreen ACT&SENSW. The organization is not financing this manuscript.

## Authors' contributions

KMF and AMB conceived and designed the study. KMF completed the ethics application, provided academic input and coordination of the study and manuscripts. JJF carried out the data collection and analysis and later drafted the manuscript. AMB provided onsite supervision, quality control and interpretation of Breastscreen records. SJ double read the pathology slides and gave input to the manuscript from a pathology perspective. All authors read and approved the final manuscript.

## Authors' information

KMF has been a breast physician since 1989 and is a Fellow of the Australasian Society of Breast Physicians. AMB is the Clinical Director of the regional breast screen program in the study and was able to provide on site guidance to JJF, who completed much of the initial work as part of a medical student research project for the Australian National University.
